# American Institutions for the Insane
*1. Of the New Hampshire State Asylum, for 1852 and 1853.2. Of the McLean Asylum, for 1853 and 1854.3. Of the Retreat at Hartford, for 1853.4. Of the Asylum for the Poor, Blackwell's Island, N. Y. City, for 1853.5. Of the Indiana State Hospital, for 1853 and 1854.6. Of the Illinois State Hospital, biennial for 1853-54.7. Of the Missouri State Asylum, biennial for 1852-53.


**Published:** 1855-10-01

**Authors:** 


### AMERICAN INSTITUTIONS FOR THE INSANE.*

1. In July, 1852, Dr. Andrew McFarland resigned the office of Superintendent of the *New Hampshire Asylum for the Insane*, having, in the language of the Trustees of the Institution, "with signal ability and devotion discharged the duties for about seven years." He was succeeded by Dr. John E. Tyler, in whom the Trustees believe that they have "a judicious, efficient, and devoted Superintendent."

The report of Dr. Tyler for the fiscal year ending on the 31st of May, 1853, seven months after he became connected with the asylum, is limited to about half a dozen pages, and contains but little matter of general interest to medical men. It is a very prudent and sensible production for a beginner.

| | Men. | Women. | Total. |
|---|---|---|---|
| Patients in the Asylum May 31, 1852 . . | 63 | 55 | 118 |
| Admitted in the course of the year . . | 68 | 64 | 132 |
| Whole number . . . . . . . | 131 | 119 | 250 |
| Discharged, including deaths . . . | 61 | 46 | 107 |
| Remaining May 31, 1853 . . . . . | 70 | 73 | 143 |
| Of those discharged, there were cured . . | 41 | 22 | 63 |
| Died . . . . . . . . . . | 5 | 3 | 8 |

Dr. Tyler complains that the number of patients is so great as to prevent a proper classification of them.

* 1. Of the New Hampshire State Asylum, for 1852 and 1853.
2. Of the McLean Asylum, for 1853 and 1854.
3. Of the Retreat at Hartford, for 1853.
4. Of the Asylum for the Poor, Blackwell's Island, N. Y. City, for 1853.
5. Of the Indiana State Hospital, for 1853 and 1854.
6. Of the Illinois State Hospital, biennial for 1853-54.
7. Of the Missouri State Asylum, biennial for 1852-53.

The report for the year ending May 31, 1854, is somewhat more extended than its immediate predecessor, but is in a great measure confined in its subjects to the *matériel* of the establishment, and to topics already familiar to our readers. The doctor complains of the pressure from without of patients, and proposes that an additional wing and a building for the violent shall be erected. "The house is now lighted with gas, and we not only find its use more convenient, comfortable, and cleanly than oil, but its brilliant light a curative means in making our previously half-lighted halls cheerful and pleasant." He says nothing of the comparative expense.

| | Men. | Women. | Total. |
|---|---|---|---|
| Patients, May 31, 1853 . . . . . . | 70 | 73 | 143 |
| Admitted in course of the year . . . . | 72 | 69 | 141 |
| Whole number . . . . . . . . | 142 | 142 | 284 |
| Discharged, including deaths . . . . | 67 | 56 | 123 |
| Remaining, May 31, 1854 . . . . . | 77* | 84* | 161 |
| Of those discharged, there were cured . . | 34 | 29 | 63 |
| Died . . . . . . . . . . . | 7 | 7 | 14 |

The whole number of patients exceeds that of any previous year by 64.

"During the whole year our household has enjoyed remarkable physical health. We have been entirely exempt from epidemics of all sorts, and acute disease has been almost unknown. Cleanliness, regularity of life, and a most healthful location have been the chief causes of this desirable state of things. The deaths which have occurred, with a single exception, were of those who for a long time had been considered incurably insane, and who at last were literally worn out by the continued and unremitting force of their malady."

| | |
|---|---|
| Patients admitted from 1843 to 31st May, 1854 . . . | 1199 |
| Cured . . . . . . . . . . . . . . | 497 |
| Died . . . . . . . . . . . . . . | 106 |

2. Dr. Bell, of the *McLean Asylum*, has written but a few reports of any length, and in the two which are now before us he is even unusually brief, both of them occupying but about a dozen pages. One reason of this brevity is mentioned in the extract which we subjoin, merely remarking that, although it may be good and sufficient for the district from which the McLean Asylum is principally supplied with patients, it is hardly equally so for many other sections of the country.

"There was a period in the history of the institutions for the insane of this country when their annual reports were looked for with an interest natural to a new topic, and when so many communities were about engaging in the great work of providing for the insane, that all information which could throw light upon the path of duty was eagerly and gratefully accepted. That time is now passed, for the demand has been essentially met, and good taste and propriety are no longer in antagonism with philanthropy, as to spreading to the world the often painful incidents connected with a sad disease and its victims. It would ever be an easy service to furnish a prolonged and interesting narrative of the cases of an asylum, were the motives now allowable which formerly justified such communications with the public."

| | Men. | Women. | Total. |
|---|---|---|---|
| Patients at the commencement of 1853 . . | ... | ... | 201 |
| Admitted in course of the year . . . . | 61 | 53 | 114 |
| Whole number . . . . . . . . | ... | ... | 315 |
| Discharged, recovered . . . . . . | 30 | 28 | 58 |
| Died . . . . . . . . . . . | 7 | 10 | 17 |
| Remaining at the close of 1853 . . . . | ... | ... | 195 |

"From a minute, kept during a portion of the year," says the report, "it is

* According to the previous figures, these should be 75 and 86.

probable that we have been obliged to refuse three times as many patients as have been received."

It will be remembered by those who have read our previous notices that Mr. Appleton gave a large fund to this institution, for the purpose of constructing apartments specially intended for persons able and willing to pay a liberal remuneration for their accommodations. The object of the donor has been partially accomplished. The "Appleton ward" for men has been completed, and in reference to its operation we find the subjoined remarks:—

"The patients themselves, in these rich and spacious quarters, can draw no unfavourable comparisons with their situations at home, and are spared one pang in the distress incident to their disease. The only drawback suggested or anticipated—that the patients who did not enjoy the new accommodations might indulge a certain sentiment of jealousy towards their more fortunate associates—has never been manifested."

Report for 1854:—

| | Men. | Women. | Total. |
|---|---|---|---|
| Patients at the commencement of the year | 94 | 101 | 195 |
| Admitted in course of the year | 70 | 50 | 120 |
| Whole number | 164 | 151 | 315 |
| Discharged, including deaths | 67 | 53 | 120 |
| Remaining at the close of the year | 97 | 98 | 195 |
| Of those discharged, there were cured | 32 | 27 | 59 |
| Died | 5 | 11 | 16 |

The McLean Asylum was opened on the 6th of October, 1828. It was under the superintendence of Dr. Wyman about sixteen years, and of his successor, Dr. Lee, two years. Dr. Bell has been the incumbent of the office since the commencement of 1837, a period of eighteen years. We believe this to be the longest term of service of any officer who has occupied an office of the kind in this country.

| | |
|---|---|
| Patients admitted under Dr. Wyman | 1122 |
| " " Dr. Lee | 189 |
| " " Dr. Bell | 2572 |
| Whole number admitted | 3783 |
| Discharged, including deaths | 3588 |
| Cured | 1802 |
| Died | 322 |

"I cannot but believe," remarks Dr. Bell, "that the time is near when the necessity of dividing this asylum, and establishing a department for one sex elsewhere in the vicinity, will result in action. The financial experience of this establishment, for many years past, would seem to demonstrate that the first outlay for such an addition to the means of treatment of those classes of the insane who are now mainly received here, would be all the demand needful upon the philanthropic and liberal of our community."

3. The last report from the *Retreat* at Hartford, Connecticut, which we passed under review, was issued during the absence of the Superintendent, Dr. Butler, upon a European tour. The one now before us bears his signature.

| | Men. | Women. | Total. |
|---|---|---|---|
| Patients in the Retreat, March 31, 1853 | 80 | 90 | 170 |
| Admitted in the course of the fiscal year | 74 | 103 | 177 |
| Whole number | 154 | 193 | 347 |
| Discharged, including deaths | 65 | 96 | 161 |
| Remaining March 31, 1854 | 89 | 97 | 186 |
| Of those discharged, there were cured | 22 | 42 | 64 |
| Died | 13 | 9 | 22 |

The Retreat was opened on the 1st of April, 1824. For ten years it was under the superintendence of Dr. Todd, six years under Dr. Fuller, and three years under Dr. Brigham. At the date of this report it has been eleven years under Dr. Butler.

| | | |
|---|---|---|
| Patients admitted in course of the term of | Dr. Todd . . | 520 |
| " " " | Dr. Fuller . . | 481 |
| " " " | Dr. Brigham . | 246 |
| " " " | Dr. Butler . | 1388 |

| | Men. | Women. | Total. |
|---|---|---|---|
| Whole number admitted . . . . . | 1266 | 1369 | 2635 |
| Cured . . . . . . . . | | | 1331 |
| Died . . . . . . . . | | | 265 |

Thus the deaths of *cases*, during thirty years, was 10·05 per cent. But, upon reference to another table, we find that the whole number of *persons* who made up these 2653 *cases* was but 1798. Of these 1798 *persons*, 248 were admitted twice each, 65 three times, 17 four times, 10 five times, 4 six times, 1 seven times, and 1 nine times. Of 1798 *persons*, 265 died, which is 14·73 per cent. The proportion of cures, upon admissions, was 50·05 per cent. But the same *person* may, in many instances, have been cured two or three times, and, in some instances, four, five, or six times. The report throws no light, even by comparison of tables, upon the number of *persons* cured. This is an imperfection which, as we have heretofore remarked, pervades nearly all the American statistics of insanity.

One further illustration, which we overlooked until the last preceding sentence was written. Dr. Butler states that the per centage of deaths on the whole number discharged since the opening of the institution is 10·82. This is correct, if calculated upon the number of *cases*. But what is the result, if otherwise regarded? Of 1798 *persons* admitted, 186 remained in the asylum. Hence, 1612 have been discharged. Of 1612 *persons* discharged, 265 died, equal to 16·43 per cent.

Of 1203 cases admitted since March 31, 1845, the age, at the time of first attack, was between 20 and 30 years in 402; between 30 and 40 years in 240. The excess of the former is equal to 66 per cent.

The causes of death in 196 cases, which have occurred since the 31st of March, 1841, were as follows: Exhaustion 36, dysentery 21, general debility 19, phthisis 14, apoplexy 12, general paralysis 12, paralysis 10, erysipelas 10, disease of the brain 9, old age 7, marasmus 7, suicide 7, "disease of lung" 5, epilepsy 4, inflammation of bowels 4, fever 3, internal hæmorrhage 3, chronic diarrhœa 3, "injury" 2, disease of heart 2, psoas abscess 1, inflammation of liver 1, disease of uterus 1, acute diarrhœa 1, dropsy 1, cancer 1.

After stating that "neither order of court, certificate of physicians, nor written application of friends or relatives" is required for getting a patient into the Retreat, and that "the admission rests solely upon the judgment of the superintendent," Dr. Butler very properly appeals to the Board of Directors to remove the responsibility from him, and place it upon the friends and the attending physician of the patient. Where are the Connecticut lawyers, judges, and legislators, that such a weak point in the barriers of the rights and liberties of the people has thus long remained unguarded?

The remarks of Dr. Butler upon the condition in which patients come to the Retreat close with this passage, which we earnestly commend to the notice and the memory of every physician in general practice: "Others, worse than all, have been brought here by the ill-judged and most pernicious means of deception, the effect of which has been, in every case that ever came under my observation, both annoying to ourselves and detrimental to the poor sufferer.

'*How can I believe* YOU, *sir*,' said a gentleman to me, while trying to soothe him, '*when these*, MY FRIENDS, *have lied to me every mile of my way here?*'"

No apology is required for making the subjoined extract, albeit somewhat longer than we are wont.

"During the six months' vacation which was so kindly granted me by the liberality of your Board, I had the pleasure of being able to visit many of the most prominent lunatic hospitals in England and Scotland. I embrace this opportunity to express my grateful sense of the cordiality and courtesy with which, as the superintendent of one of the oldest lunatic hospitals in the United States, I was everywhere received, and of the frankness and promptitude with which the details of the different institutions were shown. Every door was opened, and every department freely exhibited, evidently giving me the credit of coming to learn the advantages of their institutions, and not to seek for demerits or matters of cavil.

"My reception at some of them was more like that due to an old friend than to a stranger, and was a pleasant recognition of that kindly community of feeling which springs up in every liberalized mind towards those who are fellow-labourers in the same great commonwealth of philanthropy.

"It is evident that, from a variety of causes, a spirit of improvement is pervading these hospitals. A great impetus has of late years been given to this department of human effort, and the most beneficial and gratifying results have been attained.

"It is not expedient, in the narrow limits to which I desire to restrict this report, to go into a consideration of these causes. It is sufficient for my purpose to say that, notwithstanding a few years since our leading institutions were not surpassed by the best of theirs, it is very evident to me that we have now none which will compare with some of those lately erected there. In the older hospitals, there was manifest improvement in the buildings where original defects could never be wholly remedied. In the new institutions, those erected within a very few years, or just now going into operation, I found a beauty of structure, with a thoroughness and perfection of arrangement, which I have never seen equalled elsewhere. Among these it will not, I hope, be invidious to mention the asylums at Prestwich and Cheadle, near Manchester; at Mickleover, near Derby; at Clifton, near York; and the new asylum at Stafford.

"It was evident that in these new asylums no pains nor needful expense had been spared to obtain, in the first place, the most unexceptionable plans. The highest authorities were consulted, and their conclusions referred to the scrutiny of other practical men; the errors of preceding structures were avoided, and every improvement as readily adopted, with the single desire to obtain the best. It is evident that, generally, each succeeding structure contains improvements upon its predecessors. Once adopted, the plans have been carried out without that curtailment and distortion which sometimes, in this country, has produced such unfortunate results. In some instances, it is evident that undue expenditure has been incurred to produce external effect; but in the internal arrangements, especially, it is clear that, while in county asylums everything is plain and simple and unpretending, *that* is deemed in all the best and wisest economy which, in the long run, shall best effect the desired object.

"The chief points of excellence are extensive, well laid out, and carefully-planted airing-courts and pleasure-grounds, and sufficiency of cultivated land for out-of-door employment; spacious, airy, and well ventilated apartments; the extensive application of steam to every available purpose, cooking, pumping, heating, ventilating, &c., and open fire-places in every admissible room. The most important of all are the extensive arrangements made for the manual employment of the inmates both within doors and without. There are workshops for the different trades, in some of which these trades had been successfully taught, and in many the amount of work performed showed that the shops were sources of profit to the institution, as well as of beneficial employment to the patients.

"Another feature which struck me most pleasantly was the construction, in several hospitals, of a large and handsome room, especially for the social gatherings and amusements of the patients. My attendance at some of these festival occasions is among the most pleasant reminiscences of my visit. A large amount of profitable out-of-door labour is insisted upon in many, and the amount accomplished in some instances is highly creditable. It is very evident that, if the American institutions are to maintain the comparatively high rank to which they have justly heretofore had claim, a more liberal expenditure than has been adopted in most, in regard to occupation both of body and mind, amusement, &c., must be adopted."

4. The movement of the population of the Lunatic Asylum for the Poor of New York City, for 1853, as given in the report by Dr. Ranney, was as follows:—

| | Men. | Women. | Total. |
|---|---|---|---|
| Number of patients, January 1st | 226 | 301 | 527 |
| Admitted in course of the year | 226 | 261 | 487 |
| Whole number | 452 | 562 | 1014 |
| Discharged, including deaths | 220 | 252 | 472 |
| Remaining, Dec. 31, 1853 | 232 | 310 | 542 |
| Of those discharged, there were cured | ... | ... | 271 |
| Died | 56 | 59 | 115 |

Of those cured 14 were cases of delirium tremens, 2 of febrile delirium, and 7 were discharged, "recovered," twice each, in course of the year. These being subtracted, the number of cures is 248.

"The ratio of recoveries," remarks Dr. Ranney, "is a little more than 50 per cent. The proportion must depend much upon the length of time the disease has existed previous to admission. Usually the indigent are placed in an asylum earlier than the wealthy. For this, as well as other reasons, the percentage of recoveries in a hospital of this character should be larger than in institutions devoted to the use of the higher classes, provided the means for effective treatment be furnished."

*Causes of death*:—Consumption 45, chronic diarrhœa 14, paralysie générale 13, congestion of the brain 7, marasmus 6, typhus fever 4, typho-mania 4, paralysis 3, apoplexy 3, dysentery 3, delirium tremens 2, epilepsy 2, chronic pleurisy 2, dropsy 2, suicide 1, accidental drowning 1, pericarditis 1, hæmoptysis 1, inflammation of spinal marrow 1.

"It will be seen that consumption is the most common (cause of death). The prominent symptoms of this disease are usually absent where insanity exists. The patient will frequently walk until near the day of his death, and, if there be any cough, it is often so slight as to escape observation."

Of 3160 patients, who have been received since January, 1, 1847, no less than 2381 were foreigners, and but 779 native Americans. The largest number of natives received in any year was 149, in 1847; the smallest number, 94, in 1853. Of foreigners, the number has increased from 280 in 1847, to 393 in 1853. This is accounted for by the increase of immigration. The leading numbers in the table of nativity for 1853 are as follows:—Ireland 241, Germany 94, England 19, Scotland 10, Switzerland 5, France 4.

"Very few of the indigent insane of this city are sent to the State Asylum at Utica, and none to Flushing, Hudson, or the Bloomingdale Asylum. Either the ratio of insane is very much less among the natives, or they are kept at their homes. Probably the first supposition is true, and this may arise in part from peculiar causes incident to emigration, and in part from the shipment of the insane from Europe during a lucid interval."

Dr. Ranney, as he intimates, has had uncommon advantages for studying chronic dementia, and he asserts his belief that "by constant training, very many who, if left to themselves, fall into the most miserable condition, would become valuable aids in the asylum, even if perfect recovery did not follow. . . . If there were an important organic lesion of the brain, no great improvement could be expected; but, *from my examination of this organ in a great number of cases, the proportion in which important lesions were found has not been large. The enfeeblement of the mind depends, in many cases, upon the loss of tone, from inaction. . . . After some acute disease has existed, as mania, this organ becomes exhausted,* i. e., *loses its tone, and can only be restored by nourishment and the proper mental stimulus.*"

These remarks remind us of the case of a man of more than ordinary intellectual capacity, who was more than fifteen years a patient at Bloomingdale, a large part of the time demented. Attacked with typhoid fever, and removed to the New York Hospital, he died. The late Dr. Swett made a *post-mortem* examination, and found in the brain no lesion of importance — absolutely *nothing* whereby to explain the patient's long-continued mental incapacity.

5. In their report for 1853, the Commissioners of the Indiana Hospital for the Insane refer to the resignation of Dr. R. J. Paterson, who had held the office of superintendent from the opening of the institution, and remark that "in his departure he carried with him not only the high esteem of every other officer and attendant of the hospital, but, we trust, of every friend of the unfortunate lunatic in the State." He left on the 1st of June, 1853, and was succeeded by Dr. James S. Athon.

| | Men. | Women. | Total. |
|---|---|---|---|
| Patients in the hospital, October 31, 1852 . | 81 | 78 | 159 |
| Admitted in course of the year . . . | 74 | 82 | 156 |
| Whole number . . . . . . . . | 155 | 160 | 315 |
| Discharged, including deaths . . . | 77 | 75 | 152 |
| Remaining, October 31, 1853 . . . | 78 | 85 | 163 |
| Of those discharged, there were cured . . | 47 | 39 | 86 |
| Died . . . . . . . . . . | 7 | 7 | 14 |

"While portions of our country have suffered from disease, the Hospital for the Insane has escaped everything like an epidemic. This immunity from intermittents, remittents, and dysenteries, may be attributed to the favourable location of the institution, and to the prompt and rigid enforcement of the sanitary laws for the government of the establishment."

"Already over two hundred insane are knocking at the door of the hospital for admission, and cannot be received for want of room. The institution is crowded to its utmost capacity." The erection of an additional wing is in prospect.

Two suicides, the first which have occurred in the hospital, took place in the course of the year.

From the remarks by Dr. Athon upon the medical treatment of the insane, we make the following extract:—

"Emetics and purgatives are useful in expelling vitiated matters from the stomach and bowels. Conjoined with proper diet and exercise, they may be made subservient in restoring the natural secretions of the alimentary canal. To attempt to make a lasting and beneficial impression on the system by repeating these remedies beyond their aperient or gently evacuant effect is irrational and highly injurious to the patient. There are too many cases brought to this hospital, exhausted to mere skeletons by the use of the lancet, blisters, and purgatives, to deny, for one moment, this position. By the administration of tonics, and the use of a nutritious diet, a large proportion are restored to physical health. These remarks are made with the hope that our professional brethren who may have charge of patients before sent to the hospital, will keep in view that the *hypercynosis* system can very rarely, if at all, do good in cases of insanity."

We now come to the report for 1854:—

| | Men. | Women. | Total. |
|---|---|---|---|
| Patients in the hospital, October 31, 1853 | 78 | 85 | 163 |
| Admitted in the course of the year | 83 | 86 | 169 |
| Whole number | 161 | 171 | 332 |
| Discharged, including deaths | 88 | 84 | 172 |
| Remaining, October, 31, 1854 | 73 | 87 | 160 |
| Of those discharged, there were cured | 59 | 55 | 114 |
| Died | 5 | 8 | 13 |

*Causes of death.*—Typho-mania 3, general paralysis 2, maniacal exhaustion 2, pulmonary consumption 2, scrofula 2, tabes mesenterica 1, erysipelas 1.

"Scarcely any other disease," remarks Dr. Athon, "than what is consequent to insanity, has had a place in our wards, although the adjacent country has suffered much from summer and autumnal afflictions."

It is stated in the report of the commissioners that the great per-centage of cures "is, in part, attributable to the selection of patients, receiving those whose insanity is of the most recent date—not filling up the hospital with chronic cases that are hopelessly incurable."

Two festivals were enjoyed by the patients in the course of the year. On May-day "a banquet was given, and the day celebrated with as much parade as is usually manifested upon such an occasion;" and "the second annual celebration, at the hospital, of American independence, was attended, like the first, with the happiest results. Above a hundred patients participated in the festivities of the occasion."

| | Men. | Women. | Total. |
|---|---|---|---|
| Patients admitted from Nov. 1, 1849, to Oct. 31, 1854 | 372 | 367 | 739 |
| Discharged, recovered | 192 | 178 | 370 |
| Died | ... | ... | 63 |

Religious excitement and anxieties is alleged as the cause of the mental alienation in 61 cases, Millerism in 2, and "spiritual rappings" in 29.

"Millerism, in its day," says Dr. Athon, "startled the minds of men and turned them into religious bigots, and thence the transition was easy, in many instances, to insanity. But the spiritual rapping mania eclipses Millerism, or any other *ism,* in its agency to produce aberration of mind. The spiritualists profess to commune with departed spirits, and through their omniscience learn the condition of the dead, look into the future, and do other ridiculous things. This delusion prevails, in some parts of the country, to a most unaccountable extent, and has been prolific, beyond any other one cause, of insanity."

6. The reports from the Illinois State Hospital for the Insane are *biennial,* that which is about to occupy our attention being for the fiscal years ending on the 30th of November, 1853 and 1854.

The former superintendent having left the institution, he was succeeded, in June, 1854, by Dr. Andrew McFarland, for some years favourably known to our readers as the principal officer of the State Asylum of New Hampshire. The Trustees remark of him that their high expectations in regard to his superior qualifications for the office "have been fully realized."

| | |
|---|---|
| Patients in the asylum, Dec. 1, 1852 | 82 |
| Admitted in the two years | 265 |
| Whole number | 347 |
| Discharged, including deaths | 181 |
| Remaining, Dec. 1, 1854 | 166 |

"As the hospital affords accommodation for the two sexes equally, and the whole has been occupied the most of the time recently, the number of males and females has been nearly the same."

| | |
|---|---|
| Of the patients discharged, there were cured . | 114 |
| Died . . . . . . . . . . | 21 |

"The bodily health of the inmates of the hospital has generally been good. No epidemic has at any time prevailed, although during the past summer (1854) the danger seemed somewhat imminent from the frequency of cases of cholera in the vicinity. The instances of mortality have mainly occurred among those exhausted by long-continued mental disease. The fact cannot be questioned that most forms of mental disease shorten the period of human life. In the exceptions to the rule, in all cases of high excitement, it will be found that lucid intervals occur which allow nature to recover itself before the point of exhaustion is reached.

"Epilepsy, palsy, and consumption will annually claim a class of victims in whose temperament certain predispositions exist. But the natural termination of mania, as it usually occurs, is in a form of disease which has eluded the nomenclature of medical writers. It consists in the failure, *seriatim*, of those physical functions whose healthy performance depends upon a normal innervation. [What functions do *not* require normal innervation for normal performance?] The hesitating step and a difficulty of utterance are among the earliest indications that the brain is losing its supremacy. Calorification fails; the extremities become cold, and the individual is found hovering about the registers or other sources of artificial heat. Soon the circulation becomes enfeebled; the face assumes a swollen and stolid appearance; the extremities swell and become purple, especially if in a dependent position. Digestion becomes involved, and emaciation quickly follows. The legs break out with ulcers which soon become the nucleus of extensive sphacelation, and death is welcomed to close the scene. The individual usually sinks before all the stages in this succession of physical decay have been taken. Science has given no name to this disease, whose aspect is familiar to all who treat the associated insane. It is neither palsy, dropsy, nor marasmus, and yet it combines something of each."

Of the 406 patients who have been received at the institution since it was opened, on the 3rd of November, 1851, *only forty-six were natives of Illinois.* The others, so far as known, were immigrants from various States and countries, in the following proportions: New York 47, Kentucky 36, Pennsylvania 35, Ohio 25, Indiana 16, Tennessee 15, N. Carolina 13, Virginia 11, Vermont 7, N. Hampshire 7, N. Jersey 7, Massachusetts 5, Maryland 5, Connecticut 4, Maine 3, Delaware 3, Missouri 2, R. Island 1, Wisconsin 1, Georgia 1. Germany 30, Ireland 25, England 12, Scotland 4, France 3, Sweden 1, Russia 1, Poland 1.

Dr. McFarland thinks that, considering the great proportion of foreigners among the inhabitants of the State, the number of them in the hospital is small. "The Germans," he observes, "are the best, as they are the most numerous, of our foreign patients. They possess a healthy and elastic mental constitution; they are docile and affectionate under treatment, and grateful when they recover."

We know that for many years there has been some discrepancy of opinion among the superintending physicians of our institutions for the insane, in regard to the utility of a committee, such as is mentioned in the subjoined extract; and we rejoice that Dr. M. has found it to work so favourably:—

"Another feature in the experience of the hospital for the past year, too interesting and too important to be passed without notice, is the formation, on the part of the ladies of Jacksonville, of a benevolent association, having for its express object a regular and stated visit to the institution on the Saturday of each week, by a committee assigned in rotation. This society, appropriately styled 'The Dix Association,' we regard as a conception of the most happy kind, and its operations have been peculiarly promotive of the welfare and happiness of the unfortunate persons for whose benefit it was instituted. We earnestly hope that the zeal and faith of the society may be sustained, fully believing that its formation is an important era in the history of the institution."

The original plan of the buildings of the hospital at Jacksonville consisted of a central building and four wings, with accommodations for about four hundred patients. But two of the wings have been erected. These furnish apartments for but 168 patients, and the hospital is so much crowded that parlours are being converted into dormitories, undoubtedly to the detriment of the establishment. The question now is, whether the primary design shall be carried out, or a new hospital established in another portion of the State. The Association of Physicians to American Institutions for the Insane have deprecated hospitals intended for more than 250 patients, yet Dr. McFarland advocates the enlargement of that at Jacksonville, by the addition of the formerly contemplated wings, and alleges the reasons therefore, expressing his belief that the special circumstances render this institution an exception to the general rule.

7. The second biennial report of the Trustees and Superintendent of the Missouri State Lunatic Asylum is the first which has reached us from that institution. We have no information in regard to the precise time at which the establishment was opened, nor any detailed description of it. It appears that it contains seventy-two rooms, each designed for one patient; that it is being enlarged, so as to accommodate about seventy more; and that it is under the superintendence of Dr. T. R. H. Smith. During most of the period embraced by this report it has been over-crowded with patients, the "usual average" number having been "about one hundred;" and over seventy applications for admission have been rejected.

| | Men. | Women. | Total. |
|---|---|---|---|
| Patients in the Asylum, Nov. 29, 1852 | 34 | 28 | 62 |
| Admitted in course of two years | 68 | 55 | 123 |
| Whole number | 102 | 83 | 185 |
| Discharged, including deaths | 49 | 42 | 91 |
| Remaining, Nov. 27, 1854 | 53 | 41 | 94 |
| Of those discharged, there were cured | 20 | 23 | 43 |
| Died | 22 | 16 | 38 |

*Causes of Death.*—Epilepsy, 11; consumption, 6; chronic diarrhœa, 4; typhoid fever, 4; "ulceration of bowels," 3; paralysis, 2; "disease of heart," 2; inflammation of bowels, 2; ascites, 1; accidental burn, 1; "abscesses and gangrenous ulcers at time of admission," 1; exhaustion, 1.

"The general health of our household has been very good, excepting during the past season (1854). The intensely warm weather which continued during the summer months, in connexion with the unparalleled drought, produced an unusual amount of sickness among our patients. The citizens of Fulton, whose healthfulness, heretofore, has been proverbial throughout the State, also suffered greatly from dysentery. The prevailing disease with us was diarrhœa, with a few cases of dysentery and typhoid fever. The attacks were generally very violent in their character, and all of a typhoid type. The fatality, the number of cases considered, could not be regarded otherwise than small."

Dr. Smith, in explaining the apparently, the *really* large mortality, says:—"The patients received into this institution, when first opened, and until filled to its entire capacity, with a few exceptions, were those of long standing, who had been accumulating for many years in consequence of the want of provision for their proper treatment. A large number of these were also labouring under incurable diseases associated with their insanity." Such, or similar, has been the experience at a very considerable number of our institutions, and Dr. Smith is not the first who, at the opening of an asylum, has seen a large number of his patients succumb within the first few months.

Of the epileptics, "the majority died during convulsions, and the remainder gradually sunk under the exhausting influence of repeated attacks." The death from "accidental burn," was that of Theodore McGready, "an idiotic boy, admitted in accordance with a special act of the last Legislature. . . . A few weeks before the accident, the weather becoming cold, and in consequence of the unfinished condition of our steam heating apparatus, we were forced to take all our patients from the halls into the stove-rooms in the centre building, to prevent them from suffering. . . . Early in the morning of the day of this sad occurrence, the attendant took Theodore into the stove-room first, and returned for other patients, expecting to be absent only a minute or two. After reaching the hall, he heard him crying, ran immediately to the room, and, upon entering, to his great astonishment, found his clothes on fire. He made every effort to extinguish it, but before it could be done the burn was very severe, extending over his abdomen, his sides, between the lower extremities, the fore-arms and hands. . . . He received every attention in our power, through the day and night, but the constitutional irritation was so great that he died the next morning, about twenty hours after receiving the burn. . . . The manner in which our building is now heated (by steam) precludes the possibility of another such accident."

| | |
|---|---|
| Whole number of patients since opening of Asylum . . | 193 |
| Married 81, single 100, widows 9, widowers 3 . . . | 193 |

Insanity commenced between 20 and 30 years of age in 81; between 30 and 40 in 51. Among the assigned causes of the insanity of the patients are:—Miasmatic fevers, 25; spiritual rappings, 4.

The moral treatment pursued at this asylum is similar to that of other American institutions of the kind, and is already so fully understood as to require no further description in this place. A chapel has been constructed, and a library of 500 volumes collected by gratuitous contribution.

In his discussion of the causes of mental disorders, Dr. Smith makes the following observations:—

"It has been a source of astonishment to many that insanity should prevail to so great an extent in this highly-favoured land of ours, and seems to be increasing even in a greater ratio than our population, and is, perhaps, of more frequent occurrence in this than in most other countries of the world. The general impression is that our happy form of government . . . would be incompatible with its prevalence, at least to any great extent. It is true, the elements which enter into the composition of our government, in the abstract, seem well calculated to contribute to man's highest and best interests; yet the freedom of thought and action possessed by every individual connected with this highly-favoured state of things, the high degree of excitement incident to the different pursuits of life; the spirit of emulation; the hopes, the fears, the joys, the sorrows brought into exercise in quick succession—all tend, in a striking manner, to disturb the equilibrium so essential to the healthy action of the mental faculties; and by a repetition of the same excesses of feeling, this governing and protecting principle is lost, disease developed, and the mind in ruins one of the sad and fearful results. Is not the conclusion, therefore, justifiable, that our form of government, with the habits of our people, is calculated to increase rather than diminish the frequency of insanity, especially when we reflect that the causes referred to are acting upon ill-balanced minds and misproportioned characters, the effects of inefficiency of the intellectual and moral powers with those not favoured with good opportunities in early life, and their misdirection with those who enjoyed better advantages.

"The great practical question, then, is, What must be done in this, our happy country, already the hope and admiration of the world, to prevent, in the midst of so many exciting causes, the most terrible of all afflictions? The answer is, ample provision for, and a radical reform in, the early education of the rising generation; or, in other words, the more careful and philosophical cultivation of the intellectual and moral faculties and propensities of all, in harmony with a correct physical education. Inefficient and misdirected early education constitutes the great predisposing cause to crime as well as insanity; sad combination truly. The statistics of all hospitals for the insane prove that the great majority of the cases of insanity occur between the ages of fifteen and thirty, and the statistics of crime give us nearly the same results."

We are but little disposed to differ from our friend, Dr. Smith, in the really essential parts of this extract; but we must venture to propose some of the thoughts suggested by the perusal of it. The island of Malta was, by nature, a single mass of rock, almost wholly destitute of vegetation and of soil. Yet the Maltese boasts of his home as "The flower of the world." The Neapolitan exclaims, "See Naples and die!" (there being nothing more beautiful to be seen.) They of the country of Confucius, who claim that theirs is the "Celestial Empire," say "We have two eyes, the Europeans have one, and all the other inhabitants of the world are blind." We smile at the simplicity of the Maltese, we do not esteem Naples so much of a paradise as to be willing to die the moment we have seen it, and our people are not so much enamoured with the ocular advantages of the Chinese as to prevent a disposition to expel them from the country. Now let the impartial statesman, or jurist, or philanthropist, read the first of the two paragraphs quoted, and would he be so thoroughly convinced of our happiness, as a people, that, in the fulness of his heart, he would reiterate our expression to that effect, almost at the very beginning of the second? or would he rather pause to reflect whether, after all, we are so *truly* happy as we claim to be? We will not anticipate the decision of the question; for, most certainly, if we are not a people among the happiest in the world, it is not in default of as great a proportion of the means or elements of happiness as has ever fallen to the lot of any nation. Do we employ those means wisely? Do we combine those elements with the skill which is suggested and produced by a profound and just philosophy? Lord Morpeth, now he Earl of Carlisle, after his tour through the United States, declared it as his opinion that no other people on earth possess so many of the comforts of life as the Americans, and among none is there so little happiness.—Dr. PLINY EARLE. From the "American Journal of Medical Science" (July).

